# The Influence of Body Fat Percentage on Physiological Responses and Performance in Professional Soccer Players During a Soccer Game Simulation Protocol on a Treadmill

**DOI:** 10.3390/sports14040156

**Published:** 2026-04-15

**Authors:** Marios Hadjicharalambous, Andreas Apostolidis, Nikolaos Zaras, Eleanna Chalari, Tooba Tooba, Rabia Faiz, Omid Razi

**Affiliations:** 1 Human Performance Laboratory, School of Life & Health Sciences, University of Nicosia, Nicosia 2417, Cyprus; 2 Department of Physical Education, Ministry of Education, Sports and Youth, Nicosia 1434, Cyprus; andreasaposto@hotmail.com; 3 Department of Physical Education and Sports Science, Democritus University of Thrace, 69100 Komotini, Greece; nzaras@phyed.duth.gr; 4 Department of Sport and Physical Education, Faculty of Health Sciences, Aegean College, 10564 Athens, Greece; e.chalari@aegeancollege.gr; 5 Department of Zoology, University of Education, Bank Road Campus, Lahore 54000, Pakistan; phdaspirantresearcher@gmail.com (T.T.); rabia.faiz@ue.edu.pk (R.F.); 6 Department of Exercise Physiology, Faculty of Sport Sciences, Razi University, Kermanshah 6714414971, Iran; omid.razi.physio@gmail.com

**Keywords:** body composition, elite players, performance, physiological responses, simulating game

## Abstract

This study examined whether different body fat percentages (BF%) may influence performance, physiological responses, and fatigue in professional soccer players during a simulated soccer game protocol on a treadmill. Twenty professional male soccer players were categorized in higher (HBF%) and lower (LBF%) body fat percentage groups [HBF% > 11.5%; *n* = 11, BF% = 14.2 ± 2, LBM = 65.3 ± 8 kg, age = 22.7 ± 4 years, height = 177 ± 7 cm, weight = 76 ± 9 kg, $\dot{V}$*O*_2_*max* = 60.1 ± 4.5]; [LBF% < 11.5%, *n* = 9; BF% = 8.1 ± 1, LBM = 65.9 ± 5 kg, age = 20.1 ± 3 years, height = 179 ± 4 cm, weight = 72 ± 5 kg, $\dot{V}$*O*_2_*max* = 61.6 ± 4). Players underwent a simulated soccer game protocol on a treadmill. Cardiometabolic and hormonal responses, and fuel oxidation and performance, were evaluated. At baseline, apart from the BF% variable (*p* < 0.0001), the groups did not differ in any other physiological or physical characteristic (*p* > 0.05). There were no differences between the groups in any performance or biological parameters evaluated (*p* > 0.05), except for plasma glucose, which was higher in the HBF% group at rest and during the soccer game protocol (*p* < 0.05). In conclusion, the theory of a uniform ideal (~10 ± 2%) of BF% in elite soccer is not supported by the present study. This study suggests that when muscle mass and fitness levels of the soccer players are maintained at high levels during the competitive period, BF% represents a highly individualized characteristic rather than a uniform target across players. However, a higher BF% increased resting and exercising blood glucose concentrations, even in highly trained professional soccer players, without concomitant effects on metabolism or fuel oxidation during match play.

## 1. Introduction

Modern elite soccer is characterized by speed, agility, explosive power [1], a high level of endurance capacity [2], tactical sophistication [3,4], and strong physical [5] and cognitive preparation [6]. Players in elite soccer face substantial physical demands during matches while also coping with a congested match schedule throughout the competitive season [1,2]. Soccer-specific physical fitness elements have been associated with specific morphological characteristics in young high-level [7,8] and amateur [9] soccer players, as well as in elite male [5,10] and amateur female [11,12] soccer players. It has been reported that excess fat mass may act as a “dead weight” in activities by which the body is lifted repeatedly against gravity, such as soccer [13]. It has been previously suggested that during the game, this may negatively affect the power-to-weight ratio, acceleration and deceleration, thermoregulation, energy expenditure [14,15], explosive power, repeated sprint ability, and endurance capacity [11,16,17]. A recent study also suggested that players exceeding 20 mm in abdominal skinfold or 13% fat mass (via the Jackson and Pollock protocol) are more likely to have lower aerobic endurance during a YoYo intermittent recovery level 1 test [18]. Conversely, a higher lean muscle mass (LMM) enhances speed, power [19], and endurance [11,16,17,18] and prevents injuries [20].

However, it should be reported that proper neuromuscular activation enhances explosive strength (<200 ms) by improving nervous system efficiency, through an increased motor unit discharge rate, rather than depending only on muscle size [21,22] or body composition [23]. By optimizing motor unit recruitment, firing rates, and intermuscular coordination, the time needed to reach peak force decreases significantly [22]. This mechanism mainly relates to specific training, such as explosive and heavy-resistance strength exercises [23], and integrative neuromuscular training [24]. These adaptations are primarily neural—improving activation—and are largely independent of body composition, like muscle growth or fat loss, as they enhance neural pathways that control muscle contraction [22,23,25]. Similarly, a higher LMM induces an increase in muscular, ligament, tendon and connective tissue strength, reduces injury development by improving joint stability and shock absorption (i.e., during deceleration, stopping, or landing) and corrects muscle imbalances, which all directly mitigate the risk of muscle and joint injuries [26,27].

A lower body fat percentage (BF%) in soccer players is also associated with enhanced metabolic performance and increased distances covered with high-intensity running, allowing players to sustain high-intensity activity for longer durations [11], as reduced adiposity facilitates more efficient oxygen extraction from the blood, thereby accelerating ATP production and overall energy metabolism [17]. Players, for example, with a lower BF% have exhibited a greater reduction in muscle oxygen saturation during repeated sprints, reflecting enhanced muscle oxygen extraction and utilization, which supports more efficient energy production and sustained muscle contraction during the high-intensity phases of the game [17]. Studies have also indicated that lower BF is associated with higher metabolic power during repeated sprints in female soccer players, suggesting that players may generate metabolic substrates more efficiently, thus contributing to their ability to perform at a high level [11]. Higher adipose tissue may therefore negatively impact muscle oxygenation, potentially hindering soccer performance during high-intensity activities like sprinting [17]. However, to date, no studies have investigated cardiometabolic responses and substrate oxidation using a protocol that simulates the physiological demands of a real soccer match in professional soccer players.

Training loads and diet are the two primary drivers of body composition in soccer players, with optimal management resulting in reduced BF% (often below 10–11% in professionals) and increased LMM [28]. Regular, soccer-specific training, including high-intensity activities combined with a structured diet (high carbohydrates and moderate protein), helps lower BF throughout the competitive season [29,30]. However, a lower training load during the off-season or transition period often leads to fat accumulation [30]. Daily EE in elite soccer players is approximately 3566 ± 585 kcal [29]. During a 90 min match, elite players burn between 700 and 900 calories (kcal), but estimates may reach as high as 1300–1600+ kcal, depending on player position, body composition, and game intensity [31]. Studies reported an EE of approximately 14–15 kcal·min^−1^ over official matches in the English Premier League [32], and similar results (15.5 ± 1.8 kcal·min^−1^) were found during a simulated soccer game protocol on a treadmill [33]. However, no studies to date have examined whether different BF% in elite soccer players may influence EE and fuel oxidation during a simulated soccer game protocol on a treadmill.

One distinctive characteristic of a highly trained status in elite soccer players is an increase in LMM accompanied by a concomitant reduction in fat mass [34]. However, there is no consensus yet regarding the optimal BF% that elite soccer players should maintain during the competitive season. Studies have shown that BF% in soccer players ranges from 7% to 19% [35,36]. However, this wide range in BF% may be attributable to the use of different assessment methods across studies or to differences in fitness and skill levels among evaluated players. In elite soccer players, studies have reported the mean optimal BF% to be approximately 10% in the Premier League [16], less than 9.5% across all playing positions, including goalkeepers, in the Spanish La Liga [5], and 8.5 ± 1.7% in a squad that reached the quarterfinals of the UEFA Champions League [2]. Recently, BF% optimization in elite male players has been suggested to range between ~8% and 13%, implying a variation in BF% according to an individual player’s physiology, playing style, and field position [20]. However, in most studies reporting BF% values above 12%, goalkeepers were included in the analysis, and variations in the timing of evaluation throughout the season and the assessment methods likely contributed to these discrepancies. This approach may influence the results as goalkeepers have been reported to exhibit a relatively higher BF% compared to outfield players [37], and different evaluation methods (i.e., dual X-ray absorptiometry, air displacement plethysmography, bioelectrical impedance and skinfold calipers) [38] and the time period of the evaluation (pre-, in-, or post-season) of BF% may provide different results for the same players [2]. In addition, there is a non-scientific evidence-based recommendation that the optimal BF% of elite players should be less than 10% [20]. However, this may produce an unnecessary extra stress on players that may lead to energy deficiency during the competitive period [39] and progressively to the development of eating disorders such as restrictive energy intake or purging [40].

While testing in soccer offers valuable insights into players’ fitness level, the translation of these results to actual soccer games remains debated [41]. A common methodological limitation that studies may not entirely avoid is the evaluation of fitness parameters (i.e., power, speed, agility, strength, endurance, etc.) or physiological responses before and after training or supplementation interventions. However, no studies so far have examined physical fitness and physiological responses during a simulated soccer game protocol. Therefore, the purpose of the present study was to observe whether different BF% may influence performance, cardiometabolic responses, and fuel oxidation in professional soccer players during a soccer game simulation protocol on a treadmill. It was hypothesized that as the physical fitness and LMM of the players are consistently maintained at high levels during the competitive season, the varying, higher or lower BF% of the soccer players may not influence exercise performance or disrupt physiological and biochemical responses during the simulated soccer game protocol on a treadmill.

## 2. Materials and Methods

### 2.1. Participants and Study Approval

Twenty male, European-in-origin professional soccer players, who voluntarily agreed to participate in the current study, were divided into two groups according to their BF% [below mean group average: lower BF% (LBF%) and above mean group average: higher (HBF%)] [9]. The players were categorized as higher (HBF% > 11.5%; *n* = 11 age = 22.7 ± 4 years, height = 177 ± 7 cm, weight = 76 ± 9 kg, LMM = 65.3 ± 8 kg, BF = 14.2 ± 2%, $\dot{V}$*O*_2_*max* = 60.1 ± 4.5 mL∙kg^−1^∙min^−1^) and lower (LBF% < 11.5%, *n* = 9; age = 20.1 ± 3 years, height = 179 ± 4 cm, weight = 72 ± 5 kg, LMM = 65.9 ± 5 kg, BF = 8.1 ± 1%, $\dot{V}$*O*_2_*max* = 61.6 ± 4 mL∙kg^−1^∙min^−1^) BF% groups. This study was approved by the National Bioethics Committee (ΕΕΒΚ/ΕP/2015/20) and conformed to the World Medical Association’s Code of Ethics (the Declaration of Helsinki). The players had at least 3 years of previous professional soccer experience playing in official national league and international games. Before any evaluation, the players were informed of the tests and procedures and of the study’s nature, potential benefits, and risks. The participants gave their written informed consent and completed medical history and lifestyle questionnaires. Participants were excluded if they had any history of cardiovascular, metabolic, renal, hepatic, or musculoskeletal disorders or were taking any medication [42].

### 2.2. Anthropometric Evaluations

During the preliminary session, participants’ body weight and height were measured using a calibrated scale (Tanita Digital Scale, WB-3000, Tanita Corporation of America, Inc., Tokyo, Japan). Skinfold thickness (Harpenden caliper, British Indicators Ltd., St. Albans, UK) was measured at seven body sites, and body density and BF% were calculated as previously described [43]. All measurements were performed by the same highly experienced examiner for all players.

### 2.3. V̇O_2_max Evaluation

The participants’ maximum oxygen consumption ($\dot{V}$*O*_2_*max*) was evaluated at their 2nd visit to the laboratory. Each participant ran on a treadmill (h/p cosmos Mercury, Nussdorf, Germany) at zero incline, starting with a speed of 8 km·h^−1^, which was increased by 1 km·h^−1^ every minute until volitional fatigue. Oxygen uptake was measured breath by breath (Quark CPET, Cosmed, Rome, Italy), heart rate (HR, a Cosmed wireless HR monitor, Cosmed, Rome, Italy) was continuously assessed, and rating of perceived exertion (RPE) was recorded every 3 min using the 6–20 Borg scale [33].

### 2.4. Experimental Design

**Figure 1:**
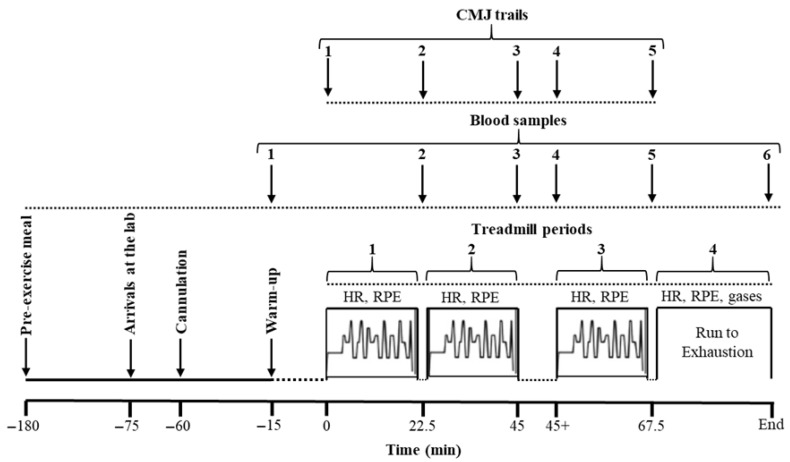
A schematic illustration of the experimental design with a diagrammatic representation of treadmill speed versus time during periods 1 to 3 of the simulated soccer game protocol. CMJ, countermovement jump; HR, heart rate; RPE, rating of perceived exertion.

Figure 1 depicts the experimental design of this study. During all tests, the laboratory temperature was set at ~21 °C [44]. The relative humidity ranged between 45% and 66%. Before the main experimental protocol, the participants underwent at least two familiarization sessions, including the full experimental procedure (the treadmill protocol, cannulation, blood sampling, RPE recording, countermovement jump (CMJ) attempts, etc.) [45].

At least 96 h after the familiarization session, participants underwent the main exercise trial, with a minimum 96 h interval between main sessions to allow full recovery [46]. To avoid diurnal variations, all fitness tests were carried out between 5:30 and 8:30 pm [47], following the consumption of a prescribed high-carbohydrate (CHO) meal, followed by a 3 h fasting period where water intake was allowed ad libitum [48]. The high-CHO meal (70% CHO, 15% fat and 15% protein, based on the participants’ diet’s characteristics; SCIENCETECH-Diet 200A software, Version II, Science Technologies, Athens, Greece) was intended to maximize liver and muscle glycogen [49]. The participants were asked to refrain from any strenuous physical activity and from alcohol and caffeine consumption for at least 48 h before the main experimental protocol [8].

The participants arrived at the laboratory 60 min before the main exercise trial, and they were seated comfortably, immersing their right hand and forearm in water at 42–44 °C for 15 min to achieve arterialization of the venous blood [33]. A 3-way function Veflon cannula (20 G) was introduced into a superficial vein on the ventral surface of the heated arm, and a resting blood sample (5 mL) was obtained. The cannula was kept patent by an infusion of 3 mL (30 IU) of heparin between samples. Before any subsequent blood sampling, 3 mL of blood was discarded to remove any heparin residues from the vein. Following 15 min of warm-up, including running, skipping, jumping, and stretching [48], the participants performed two CMJs (OptoJump Next, Microgate, Bolzano, Italy) with an arm swing, starting from the standing position. During the CMJs, the participants were asked to jump vertically and land with both feet simultaneously to measure the best jump height. Between jumps, 30 s of recovery was allowed. Following this first battery of CMJs, the participants started performing an intermittent treadmill protocol aimed at simulating a soccer game based on Drust et al. [50], slightly modified by Apostolidis et al. [33]. The protocol consisted of three 22.5 min periods, followed by a fourth period, which included running to exhaustion. The treadmill incline was set at zero during the whole treadmill protocol, whereas the speed of the treadmill was periodically set at 6, 12, 15, 18, or 21 km·h^−1^ (Figure 1). During each period, the participants ran at 6 km·h^−1^ for 60 s, at 12 and 15 km·h^−1^ for 50 s each, at 18 km·h^−1^ for 10 s, and at 21 km·h^−1^ for 25 s, the remaining time being spent in transition from one speed to the next.

At the end of the first running period of 22.5 min, the 2nd blood sampling was obtained, and the 2nd battery of the CMJ test was performed, as before exercise, within 5 min. This was repeated at the end of the second running period (the 3rd blood sampling and CMJ test at the end of the first half), which was separated from the third one by 15 min, corresponding to the interval between half-times in a soccer game. The 4th blood sampling was obtained (immediately before the initiation of the second half), and the 4th battery of the CMJ test was repeated right before and after the third treadmill period. Immediately following that, the participants were connected to a gas analyzer by putting on a face mask and embarked on the fourth period, consisting of running at a speed corresponding to 75% of $\dot{V}$*O*_2_*max* to volitional exhaustion. HR was continuously recorded, and RPE was determined at the beginning, middle, and end of each period of the treadmill protocol.

### 2.5. Energy Expenditure and Substrate Oxidation

Oxygen uptake ($\dot{V}$*O*_2_, L·min^−1^), carbon dioxide production ($\dot{V}$*CO*_2_, L·min^−1^), and the respiratory exchange ratio (RER) were measured continuously during the first 5 min of the fourth period [33]. Energy expenditure (EE) and rates of fat and carbohydrate oxidation were estimated as follows. Initially, raw breath-by-breath data were examined for outliers, and any breaths with $\dot{V}$*O*_2_, $\dot{V}$*CO*_2_, and RER values that lay more than four standard deviations away from the mean response were excluded [33]. Then, EE (kcal·min^−1^) was calculated using the following equation [33]:

$$\mathrm{Energy}\;\mathrm{expenditure}=\{4.686+[(\mathrm{RER}-0.707)/0.293]\times 0.361\}\times \dot{V}O_{2}$$

Additionally, the rates of fat and carbohydrate oxidation (g·min^−1^) were calculated using the following equations [33]:

$$\mathrm{Fat}\mathrm{oxidation}=(1.67\times \dot{V}O_{2})-(1.67\times \dot{V}{CO}_{2})$$

$$\mathrm{Carbohydrate}\mathrm{oxidation}=(4.55\times \dot{V}{CO}_{2})-(3.21\times \dot{V}O_{2})$$

### 2.6. Blood Treatment and Biochemical Analyses

Blood samples were immediately dispensed into K_2_EDTA tubes and centrifuged at 4000 rpm for 10 min to obtain plasma. Plasma samples were stored at −80 °C until further analysis for glucose, glycerol, NEFA (Greiner Bio-One, Frickenhausen, Germany), and lactate (DiaSys Diagnostic Systems, Holzheim, Germany), using standard enzymatic methods in an automatic spectrophotometric analyzer (COBAS MIRA Plus CC, Roche Diagnostics Systems, F. Hoffmann-La Roche Ltd., Basel, Switzerland) [33]. Epinephrine was measured (Tecan Group Ltd., Mannedorf, Switzerland) using a microplate absorbance reader (Tecan Sunrise with ELISA microplate reader, Tecan Austria GmbH, Grödig, Austria) [33].

### 2.7. Statistical Analysis

A post hoc (G* Power, 3.1.9.7: F tests, repeated-measures ANOVA, and between–within factors) sample power analysis was performed, revealing an actual power of 0.88 (effect size f = 0.25, α err prob = 0.05) for a maximum number of 20 participants (÷by 2 groups) for the differences between the groups. The SPSS statistical package (version 28 for Windows^®^) was used for all the statistical computations (SPSS Inc., Chicago, IL, USA). All data are expressed as the mean ± standard deviation (SD) following the normality of distribution test (Shapiro–Wilk test). Baseline physiological characteristics of the two groups were compared by an independent sample *t*-test (Table 1). All test variables were analyzed by GLM repeated-measures ANOVA (one between and one within factor). Partial eta-squared (*η*^2^) was used to report effect sizes (ESs), categorized as small (η^2^ < 0.06) and large (η^2^ > 0.14) [51]. Statistical significance was declared at *p* ≤ 0.05.

## 3. Results

### 3.1. Physical and Physiological Characteristics

The groups did not differ in any of the evaluated variables (*p* > 0.05) except for BF% (*p* < 0.001) (Table 1).

### 3.2. Physical Fitness and Performance

**Figure 2:**
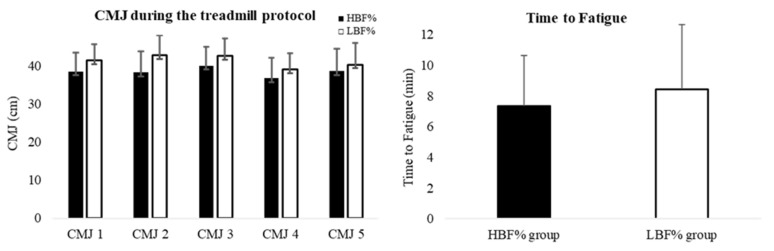
Mean and SD of CMJ (**left**) [at baseline (CMJ1) and during the treadmill protocol (CMJ2 at 22.5 min; CMJ3 at 45 min; CMJ4 after half-time, immediately before 2nd half; and CMJ5 at 67.5 min)] and time to fatigue (**right**) in higher (black columns) and lower (white columns) body fat percentage (BF%) groups. CMJ: Countermovement jump.

There was no significant difference in CMJs [F_1,18_ = 1.67, *p* = 0.213, ES = 0.085, 95% CI: −7.44, 1.77] and time to fatigue (*p* = 0.53, ES = −0.32, 95% CI: −4.89, 2.41) between HBF% (7.4 ± 3 min) and LBF% (8.4 ± 4 min) groups (Figure 2).

### 3.3. Blood Metabolites

**Figure 3:**
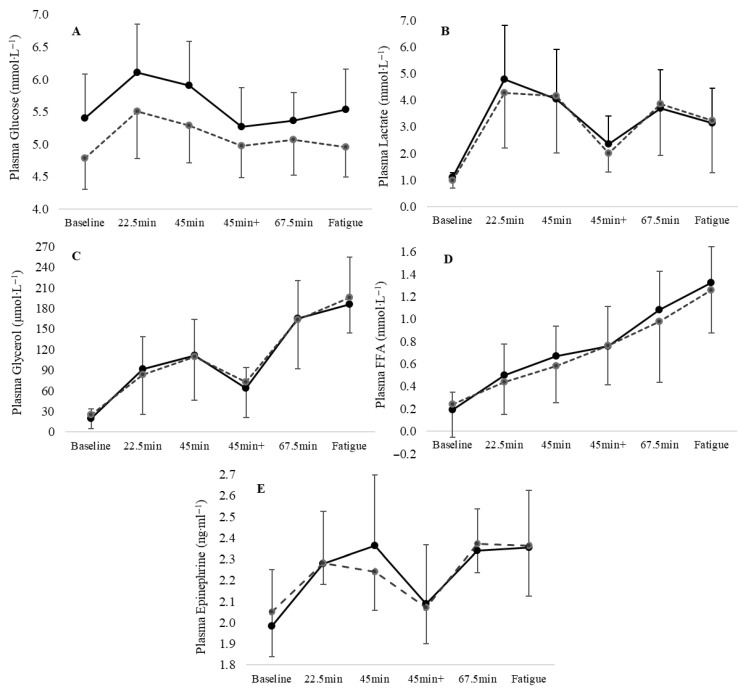
The mean and SD of plasma (**A**) glucose, (**B**) lactate, (**C**) glycerol, (**D**) free fatty acids (FFAs), and (**E**) epinephrine in higher (solid lines) and lower (dashed lines) BF% groups at the 6 blood samplings, at rest, and during the treadmill protocol.

Plasma glucose (Figure 3) exhibited significant group [F_1,18_ = 8.40, *p* = 0.01, ES = 0.32, 95% CI: 2.48, 15.52] and time [F_5,90_ = 6.35, *p* < 0.001, ES = 0.39, 95% CI: 0.11, 13.29] main effects. Plasma glucose was higher in the HBF% group at baseline (*p* = 0.035, ES = 1.0, 95% CI: 0.85, 21.37) and remained elevated throughout the running protocol, reaching significant differences at the end of the first half (45 min, *p* = 0.044, ES = 0.98, 95% CI: 0.36, 21.96) and at the time to fatigue (*p* = 0.033, ES = 1.0, 95% CI: 0.97, 19.95). There were no significant differences between the groups in plasma lactate [F_1,18_ = 0.032, *p* = 0.86, ES = 0.002, 95% CI: −1.14, 1.35], glycerol [F_1,18_ = 0.018, *p* = 0.919, ES = 0.001, 95% CI: −39.99, 36.26], FFA [F_1,18_ = 0.008, *p* = 0.93, ES = 0.001, 95% CI: −45.19, 41.54], and epinephrine concentrations [F_1,18_ = 0.003, *p* = 0.954, ES = 0.001, 95% CI: −0.183, 0.194] (Figure 3).

### 3.4. Cardiovascular Responses, RPE, EE, and Fuel Oxidation

There were no significant differences between the groups in the average HR [F_1,18_ = 0.463, *p* = 0.50, ES = 0.025, 95% CI: −7.06, 13.82] and RPE [F_1,18_ = 0.982, *p* = 0.34, ES = 0.051, 95% CI: −0.70, 1.96] of each of the four periods of the treadmill protocol. No differences were also observed in RER and EE and in fat and CHO oxidation (*p* > 0.05). The overall RER and EE for HBF% and LBF% were 0.82 ± 0.05 and 0.82 ± 0.049 kcal·min^−1^ and 16.4 ± 2.6 and 17.6 ± 1.7 kcal·min^−1^, respectively. The overall CHO and fat oxidation for HBF% and LBF% were 1.7 ± 0.8 g·min^−1^ and 1.8 ± 0.7 g·min^−1^ and 0.96 ± 0.3 g·min^−1^ and 1.05 ± 0.3 g·min^−1^, respectively.

## 4. Discussion

The aim of the current study was to examine the effect of BF% on soccer-specific performance, cardiometabolic and biochemical responses, fuel oxidation, and time to fatigue in professional soccer players during the simulation of a soccer game protocol on a treadmill. The prototype approach of the current study was to evaluate soccer-specific physical fitness and biological responses during a soccer game simulation protocol on a treadmill imitating a real soccer game scenario. To the authors’ knowledge, this is the first study that provides physical and biological evidence of the actual effect of BF% in professional soccer players during a simulated soccer game. The current study suggests that BF% is an individual condition of each particular player. A relatively higher value (~14.2%) than the typical values suggested as the optimal (~10 ± 2%) BF% in elite soccer does not seem to negatively influence soccer-specific performance, metabolism, fuel oxidation, and time to fatigue during the soccer game simulation protocol on a treadmill. However, a higher BF% significantly increases resting and exercising blood glucose levels without influencing glucose metabolism. This may suggest a negative influence of blood glucose regulation at rest and during exercise, even in elite soccer players. It is noted that due to the small sample size, the current results should be interpreted with caution.

Previous studies have shown that the ideal BF% of elite soccer players ranges between 8% and 12%, with an average of ~10% in the Premier League [16] and even lower than 9% in the Champions League [2,3,4]. These values suggest higher EE demands during training sessions and official games due to the higher intensity of training and game demands, elements that characterize soccer players at the elite level. During match play, excess fat mass has been suggested to act as dead weight for the players and negatively impact players’ explosiveness, repeated sprint performance [13,16], power-to-weight ratio, thermoregulatory efficiency, acceleration and deceleration capabilities, energy expenditure [14,15], and endurance capacity [11,16,17,18]. In addition, a lower BF% has been associated with improved muscle oxygen utilization and energy production, especially during high-intensity activities like sprinting, suggesting that active muscles may extract and use oxygen more efficiently, which is crucial for sustained high-intensity performance during a soccer game [22]. In the current study, a specific repeated sprint ability test was not performed, and acceleration, deceleration, and thermoregulation were not evaluated. However, RPE during the repeated regular high-intensity running (i.e., the speed of the treadmill was periodically set at 6, 12, 15, 18, or 21 km·h^−1^), neuromuscular explosiveness, endurance capacity, and fuel oxidation were not different between the groups. Consequently, the current study suggests that when the LMM and cardiorespiratory capacity of the players are maintained at high levels during the competitive period, without exhibiting differences between the evaluated groups, the significantly different BF%, as observed between the groups at baseline, does not seem to have a meaningful negative influence on crucial fitness and metabolic components (i.e., neuromuscular explosiveness, endurance capability, RPE, EE and fuel oxidation) of the soccer players during a demanding soccer game. Supporting the current findings, previous studies have indicated that a high LMM is important for the production of speed, strength, and power during match play [18] and that training programs aimed at increasing LMM may improve both aerobic and anaerobic performance [17,52,53].

It should be noted that during the current treadmill protocol, maximum running speed did not exceed 21 km/h. This speed does not reflect the speeds that some players can reach during official matches, where the average peak speed is around 30.7 km/h, with defenders and forwards often faster than midfielders (e.g., 33.03 ± 1.35 km/h for forwards) [3,4,54,55]. Similarly, ball play could not be incorporated during this simulation soccer game protocol, and it does not fully mimic real match conditions. However, in the current study, there were no interruptions in running activities due to fouls, free kicks, VAR checks, cards, injuries, etc., in which players might stop even walking, allowing them to fully recover from high intermittent speeds, power, and endurance efforts. Therefore, although this protocol does not exactly replicate a real soccer game, especially regarding higher speeds and ball play, it could physically stress players much like a real match. This is supported by their average RPE scores, which were 16 ± 1.5 on the Borg 6–20 scale, in both groups, during the intermittent running protocol, reaching 20 at the end of the fatigue phase. The current findings align with previous research, indicating that the average RPE on the Borg 6–20 scale for elite soccer players during a 90 min competitive match generally falls between 14 and 17 [56].

In the current study, plasma glucose was significantly higher at rest and during exercise in the HBF% group, with a large ES, signifying the relationship between BF and blood glucose metabolism. However, this difference in blood glucose did not influence either fuel oxidation or exercise performance. The elevated plasma glucose observed in the HBF% group may reflect subtle alterations in glucose handling associated with higher BF%, which may affect insulin sensitivity and glucose regulation [57], although plasma insulin and insulin sensitivity were not directly assessed in the present study. For example, previous studies have shown a positive correlation between BF% and blood glucose levels and metabolism, as a higher BF% is associated with increased insulin resistance, which disrupts glucose regulation in sedentary adolescents [58], adults [59,60,61,62], athletes [63], and animal models [64]. Individuals with relatively high BF, particularly abdominal fat, are therefore more likely to exhibit insulin resistance, in which cells become less responsive to insulin, resulting in elevated blood glucose levels [60,65,66,67]. While athletes generally have a significantly lower overall BF% compared to the general population, individual variations in body composition may still influence their blood glucose levels [59,66]. In the current study, skeletal muscle mass was not different between the groups. However, although increased skeletal muscle mass can enhance glucose uptake and utilization, potentially lowering blood glucose levels [59,68], this effect was not evident in the observed players with higher BF%. Consequently, even in elite athletes, reducing BF%, ideally to below 11.5%, may be necessary to elicit meaningful reductions in both resting and exercise-induced blood glucose levels for health purposes. However, when planning to reduce BF, specific precautions should be taken into consideration to prevent the loss of LMM, as such reductions may negatively impact soccer performance, including speed, power, agility, and endurance [2,16,19,52,53]. The optimal period for achieving favorable adaptations in body composition is the pre-seasonal period [8,69], since fluctuations in body composition during the in-season period may negatively influence the physical fitness of the players and the overall game performance [5,11,17,20,70,71].

Plasma lactate, glycerol, FAA, and epinephrine concentrations, as well as RER, EE, CHO and fat oxidations, were not different between the groups. The metabolic rate of all the above-mentioned evaluated variables was identical in both groups at baseline and during the game protocol. To the authors’ knowledge, this is the first study that has directly examined the influence of BF% in metabolic responses and fuel oxidation during a protocol that imitates a real-time soccer game scenario in association with soccer performance, subjective fatigue effort, and time to fatigue. Consequently, the potential influence of BF% on metabolism and substrate utilization during a soccer match, comparing players with relatively higher (>12%) and lower (<10%) BF%, remains unexplored. In the general population, fat oxidation capacity is directly associated with body fatness, and the increase in fat oxidation in response to submaximal exercise appears to be attenuated in individuals with obesity [72]. On the contrary, other authors have observed that there is no influence of excess BF% on lipid oxidation during exercise [73]. Similarly, in the current study, a higher BF% did not influence EE, fuel oxidation, and epinephrine regulation during the exercise protocol, suggesting that when the muscle mass and endurance capacity of the players are not different, the relatively excess BF% does not influence RER, EE, fuel oxidation and epinephrine regulation during high-intensity exercise, such as in a soccer game. Thus, since plasma epinephrine concentration and fuel oxidation were not different between the groups, during the soccer game protocol, plasma lactate, glycerol, and FFAs were also not expected to differ.

In young high-level soccer players [7,8,74] and female futsal players [75], lower body fat is associated with greater endurance capacity, enabling players to better maintain their performance throughout a match. This is likely because carrying excess body fat increases the energy cost of movement, which may lead to accelerating the onset of fatigue [75]. Similarly, a lower BF% in young amateur players [7,9], adult semi-professional players [76], and female futsal players [75] has been associated with enhanced performance in explosive tasks, such as jump performance, speed, quickness, agility, and ball-kicking speed during a soccer game. However, in the current study, physical fitness measures such as CMJs and endurance capacity, as well as RPE, time to fatigue, RER, EE, CHO, and fat oxidation, were not different between the HBF% and LBF% groups. Reinforcing our previous conclusion, these results might be due to similar LMM observed between the groups. Previous studies, for example, suggested that excess body fat may reduce a player’s explosive ability, while a higher amount of lean muscle mass is associated with greater power production for actions like kicking, jumping, and endurance capacity [7,8,9,74,75,76]. However, in the current study, since LMM as well as hormonal, metabolic (except for plasma glucose), fuel oxidation, and RPE did not differ between groups, differences in explosive performance and endurance capacity, as they were evaluated during the game, were not expected. Consequently, in well-trained, professional soccer players, when fat-free muscle mass is maintained at a high level, a relatively higher value than 11.5% of BF% does not limit soccer-specific performance, such as sprinting and jumping, as well as endurance capacity and fuel oxidation during the game. The current findings support Collins et al. [20], who suggested that in elite soccer, there is no uniform approach regarding players’ BF% and the ideal physique, considering both BF and LMM levels, which vary according to an individual player’s physiology, playing style, and field position.

### Study Limitations

Several important limitations of the current study should be addressed. (1) Body composition varies between players and is influenced by factors like playing position, age, and individual body type. For example, wingers often have a lower BF%, while other field positions might benefit from slightly higher levels of LMM for physical contests. In the current study, the small sample size precluded the stratification of participants by playing position. (2) Even if the participants of the current study were all elite professionals, due to the small sample size, the results should be interpreted with caution. (3) Due to the nature of the protocol, repeated sprint ability, agility performance, and soccer-specific technical skills with and without the ball, which are all key fitness components in elite soccer, were not assessed in the current study. (4) We did not control for scars and tattoos of the players, which may influence sleep, aberrant myotatic reflexes, lymphatic drainage, the immune system, and potentially exercise performance [77,78,79,80].

## 5. Conclusions

The non-scientific, evidence-based target of less than 10% body fat, as well as the idea that the ideal body fat percentage in elite soccer should be between 8 and 12%, is not supported by the current study. The current findings suggest that, from a performance point of view, provided LMM and cardiorespiratory fitness are maintained at high levels during the competitive period, BF% should be considered an individual characteristic, varying according to each player’s unique physiology, and there is no rigid threshold for BF%. However, a higher than 11.5% BF% may elevate resting and exercising blood glucose levels, even in professional soccer players, without influencing fuel oxidation. Consequently, for glycemic control in a health context, values <11.5% and, if possible, closer to ~8.1 ± 1% are recommended. The current study enhanced our understanding of how body fatness affects physiological responses and performance in elite soccer during matches. From a practical viewpoint, this study emphasizes the importance of an individualized approach rather than a strict generic recommendation concerning the optimal BF% of elite players. Future studies may examine the effects of BF% on performance and biological responses in elite soccer by grouping players by playing position for both males and females.

## Figures and Tables

**Table 1:** Physical and physiological characteristics of the groups.

Physical and Physiological Characteristics	LBF% Group (*n* = 9)	HBF% Group (*n* = 11)	*p*-Values	95% CI
Age (years)	20.1 ± 3	22.7 ± 4	0.153	−0.73, 5.9
Body mass (kg)	72 ± 5	76 ± 9	0.232	−2.96, 11.42
Body height (cm)	179 ± 4	177 ± 7	0.642	−6.43, 4.07
BF%	8.1 ± 1	14.2 ± 2 *	0.001	3.74, 7.87
LMM (kg)	65.9 ± 5	65.3 ± 8	0.821	−7.0, 5.6
$\dot{V}$*O*_2_*max* (mL·kg^−1^·min^−1^)	61.6 ± 4	60.1 ± 4.5	0.447	−5.61, 2.58

Values are means ± SD. *: a significant difference between the groups. BF%: body fat percentage; LMM: lean muscle mass.

## Data Availability

The data that support the findings of this study are available from the corresponding author (M.H.) upon reasonable request.

## Abbreviations

The following abbreviations are used in this manuscript:

BF%	Body fat percentage
HBF%	Higher body fat percentage group
LBF%	Lower body fat percentage group
LMM	Lean muscle mass
ATP	Adenosine triphosphate
$\dot{V}$*O*_2_*max*	Maximum oxygen uptake
HR	Heart rate
RPE	Rating of perceived exertion
CMJ	Countermovement jump
CHO	Carbohydrate
$\dot{V}$*O*_2_	Oxygen uptake
$\dot{V}$*CO*_2_	Carbon dioxide production
EE	Energy expenditure
RER	Respiratory exchange ratio
